# Instant Biofeedback Provided by Wearable Sensor Technology Can Help to Optimize Exercise and Prevent Injury and Overuse

**DOI:** 10.3389/fphys.2017.00167

**Published:** 2017-04-03

**Authors:** Peter Düking, Hans-Christer Holmberg, Billy Sperlich

**Affiliations:** ^1^Integrative and Experimental Exercise Science, Institute for Sport Sciences, Julius-Maximilians UniversityWürzburg, Germany; ^2^Swedish Winter Sports Research Centre, Mid Sweden UniversityÖstersund, Sweden; ^3^Department of Physiology and Pharmacology, Karolinska InstituteStockholm, Sweden; ^4^School of Sport Sciences, UiT Arctic University of NorwayTromsø, Norway

**Keywords:** performance monitoring, health monitoring, sports technology, coaching, training optimization

With great interest, we have been following the developing variety and popularity of commercially available wearable sensor technologies, as well as the discussion concerning their usefulness for improving fitness and health (Duking et al., 2016; Halson et al., 2016; Sperlich and Holmberg, 2016). Although many of these devices may not necessarily fulfill scientific criteria for quality (Sperlich and Holmberg, 2016) or may pose a threat to the security of personal data (Austen, 2015), we would like to emphasize here that many individuals who seek to improve their health or physical performance do so on their own, without the guidance of professionals to design their fitness training. Although professional guidance is, of course, important, such individuals and, especially beginners, would find instantaneous (bio)feedback beneficial for optimal adaptation and prevention of overuse or injury. We believe wearable sensor technologies, in conjunction with appropriate (mobile) applications, data mining and machine learning algorithms, can provide biofeedback that is useful in many ways.

In this context, biofeedback is considered to be individual data related to the body (e.g., heart rate and motion, including acceleration of body segments and much more). Such biofeedback, provided either haptically, audibly and/or visually, can augment or even replace a sensory organ, allowing the individual to react appropriately (Fuss, 2014). For example, visual biofeedback provided by wearable sensors can help modulate gait in a manner that reduces loading of the legs while running, thereby lowering the risk for stress fracture of the tibia (Crowell and Davis, 2011).

Current and ongoing improvements in wearable sensor technologies and their applications provide vibrotactical biofeedback (Afzal et al., 2016) and/or auditory signals through so-called “(h)earables” or other types of receivers. Visual biofeedback may be given by smartwatches and/or –phones and in the near future by smart glasses or contact lenses (Hosseini et al., 2014). We believe that such easily accessible biofeedback from wearable sensors that are (i) unobtrusive and do no harm, (ii) reliable and valid, and (iii) provide relevant information can help individuals make their training more effective.

Clearly, objective biofeedback provided by wearable sensors can reveal aspects of an individual's health and training, which simply cannot be otherwise accessed. Examples include neuromuscular fatigue and forces acting upon the cruciate ligaments (Belbasis et al., 2015), certain aspects of a soccer player's kicking technique (Weizman and Fuss, 2015), metabolites and electrolytes in sweat (Anastasova et al., 2017), and hydration status and shifts of fluid in the body (Villa et al., 2016). In addition, many other types of monitoring are presently under development.

To summarize, we believe that the provision of haptic, audible and/or visual biofeedback by high-quality wearable sensors in connection with data mining and machine learning algorithms will assist athletes, especially beginners, in optimizing their training and health by helping to prevent overuse and injury.

## Author contributions

All authors listed have made substantial, direct and intellectual contribution to this work and approved it for publication.

### Conflict of interest statement

The authors declare that the research was conducted in the absence of any commercial or financial relationships that could be construed as a potential conflict of interest.
